# The relationship between perceived teacher support and student engagement among higher vocational students: A moderated mediation model

**DOI:** 10.3389/fpsyg.2023.1116932

**Published:** 2023-02-17

**Authors:** Xiuni Xu, Zhen Wu, Dongpo Wei

**Affiliations:** ^1^School of Automation and Electrical Engineering, Tianjin University of Technology and Education, Tianjin, China; ^2^School of Electrical Engineering, Longdong University, Qingyang, Gansu, China; ^3^School of Vocational Education, Tianjin University of Technology and Education, Tianjin, China; ^4^School of Mechanical Engineering, Shandong Huayu University of Technology, Dezhou, Shandong, China

**Keywords:** perceived teacher support, student engagement, optimistic attributional styles for explaining positive events, learning drive, mediating effect, moderation effect

## Abstract

**Introduction:**

Student engagement is a best predictor variable of student’ development and success. It can be highly influenced by internal and external environmental factors, such as perceived teacher support.

**Methods:**

In order to explore the influence of perceived teacher support on student engagement among higher vocational students, this study conducted a questionnaire on 1,136 Chinese higher vocational students using perceived teacher support, basic psychological needs satisfaction, learning drive, student engagement and Optimistic attributional styles for explaining positive events (OAS_P) five scales.

**Results:**

The results show that: (1) Perceived teacher support can’t indirectly predict the student engagement among higher vocational students through basic psychological needs satisfaction; (2) Perceived teacher support can indirectly predict student engagement through learning drive; (3) Perceived teacher support can indirectly predict student engagement through basic psychological needs satisfaction and learning drive; (4) OAS_P has a significant moderation effect on both learning drive and student engagement.

**Discussion:**

The finding of this study stated that perceived teacher support has a significant influence on student engagement. So in the teaching process, teachers should pay attention to their learning psychology, provide them with various support and encouragement and beneficial guidance, stimulate their learning drive, help them form a positive and optimistic attribution style, and make them actively participate in learning and school life.

## 1. Introduction

According to the statistical data released in The Main Results of National Education Statistics in (2021), higher vocational colleges (junior college) enrolled 5,525,800 college students, which accounted for 55.2% of the total enrollment of higher education, and the scale of higher vocational education is constantly developing and growing. Also, the higher vocational colleges admission methods are diversified (Yang et al., 2015), such as unified college entrance examination, middle and higher vocational colleges through enrollment and expand enrollment (former ordinary high school graduates, secondary vocational graduates and social candidates including migrant workers, laid-off workers, veterans, new professional farmers, etc.), which makes the structure of higher vocational students is complex. However, under the influence of traditional ideas, compared with ordinary higher education, vocational education still lacks enough social attraction, and has fewer excellent students. Many higher vocational students have a weak foundation, insufficient learning ability, poor independent ability, lack of learning interest and internal motivation, and lack of student engagement. In 2020, the Research Institute of Vocational and Technical Education Center of Chinese Ministry of Education conducted the first national survey on vocational colleges, the findings showed that there were many problems for higher vocational students’ learning, such as lack of active learning initiative in and out of class, and relative lack of deep cognition, the student engagement is not satisfactory (Cai and Wang, 2021). According to the constructivist learning theory, learning is an active and student-centered process in which students are active learners, and they actively “construct” knowledge. Student engagement refers to students’ active participation in learning tasks and activities (Fredricks et al., 2004), it is an active and positive psychological state related to learning. It’s an important indicator to measure the quality of students’ learning process, and it is highly correlated to students’ learning sustainability, academic satisfaction, learning performance and academic completion (Li and Li, 2021), it is the best predictor variable of student’ development and success. Student engagement can not only effectively predict students’ current academic performance, but also vertically predict students’ learning and work achievement 10 years later, it has a profound impact on the development and growth of students (Jia et al., 2020), so it is of great value for students’ learning and life. Therefore, it’s a promising avenue to make higher vocational students proactively and actively engaged in the learning process, and then ameliorate academic performance.

The importance of student engagement is beyond doubt. But its specific connotation has caused many debates. Referring to student engagement, different scholars have different views. In this study, we adopted the conceptual model of student engagement of Fredricks et al. (2004), which is widely used. They described student engagement as a student commitment or investment in school and school activities, considering student engagement as three distinct but related multi-dimensional concepts, including behavioral engagement, cognitive engagement and emotional engagement. Behavioral engagement refers to students’ behaviors that are directly related to the learning process, observable and consistent with desired learning behaviors, such as, effort, active participation in discussions, and participation in curriculum and extra-curricular activities. Cognitive engagement refers to cognitive energy investment used by students for learning, understanding and mastering knowledge and skills, involving the effective use of self-regulated meta-cognitive strategies and deep learning strategies; emotional engagement refers to the affections and emotions of students in learning activities, such as enthusiasm, interest, enjoyment, boredom or anxiety.

Student engagement is not a fixed but a plastic existence state which is highly influenced by internal and external environmental factors. According to the nested environmental structure influencing individual achievement at different levels in Bronfenbrenner ecosystem theory, the microsystems may be the most significant factors for children’s participation in learning (Bronfenbrenner, 1977). School is the direct learning environment in addition to family, and it is a closest micro-system affecting student development. In this micro-system, teachers are the “authoritative figures” that students have the most contact with, and teachers have a great influence on their students (Chen and Yang, 2009). Teacher support is an important source of social support, and it is a positive social resource. This study adopted self-determination theory’s (Deci and Ryan, 2000; Ryan and Deci, 2000) classification of teacher support. Teacher support is divided into autonomy support, emotion support and competency support. Autonomy support is to regarding students as autonomous and decision-making capable individuals, providing them with opportunities and choices for decision-making, respect for students’ independent choice, and focus on students’ interest and focus in teaching process. Emotion support means that teachers care for students, consider for them, and provide help to students when necessary (Connell and Wellborn, 1991). Competency support directly corresponds to the satisfaction of students’ competency needs, namely, the teacher raises clear expectations to help students develop, provides guidance, help, and encourages students to believe that they can successfully participate in a task and provide feedback on academic performance. Subjective perception of teacher support affects learning more directly than objective teacher support itself. Maybe student engagement of higher vocational students will be affected by the perceived teacher support. So, in this study, we try to explore the relationship between perceived teacher support and student engagement among higher vocational students through empirical research methods, and illuminating the “black box” of perceived teacher support and student engagement, so as to find the focus to improve higher vocational students’ student engagement.

## 2. Literature review and research hypotheses

### 2.1. Perceived teacher support and student engagement

In learning process, students need teachers to give all aspects of encouragement, support and beneficial guidance. When students feel teachers’ care and love for themselves, and guidance or help for their academic or life problems, students will show positive learning behaviors and achieve good academic performance. Many studies have confirmed that students with perceived teacher support can be more confident, more active, and more persistent in learning. Fraser and Fisher (1982) showed that students feeling emotionally supported by teachers were more likely to experience motivation for mastery, learning enjoyment, and academic success, and exhibit behaviors in task. Students who think their teachers support them show more adaptive functions in school than those who don’t (Ahmed et al., 2010). And Fall and Roberts (2012) had found that perceived teacher support can positively predict student engagement among middle school students. Will perceived teacher support predict student engagement among higher vocational students? So, we hypothesize that perceived teacher support can directly affect student engagement among higher vocational students.

### 2.2. Basic psychological needs satisfaction as a mediator

According to Self-determination theory (SDT), individuals are born with three basic psychological needs: autonomy, competence and relatedness. These three types of basic psychological needs are not only the innate, universal and basic psychological needs of human beings, but also are developed and satisfied in a social environment and with interpersonal and institutional support (Deci and Ryan, 2002). Brown and Ryan (2006) believed that needs satisfaction was a dynamic, plastic experience, and vulnerable to social environment changes. Teachers’ autonomy supports can positively predict basic needs satisfaction, and the basic needs satisfaction can positively predict the autonomous motivation, and then predict positive emotions and student engagement (Kaplan, 2018). Considering the relatedness which students require, teachers are suggested to provide emotion support to satisfy their emotional needs. Emotion support is characterized by teachers’ concern for students’ wellbeing, personal needs, adequate feedback, and students’ perception of the warmth, trust, and respect from teachers (Gj and Yw, 2019). When it happens, an affective and close teacher-student relationship will result in. A good teacher-student relationship promotes students’ sense of security, which not only lays a good foundation for their academic participation, but also leads to positive mood changes. A good teacher-student relationship encourages students to participate in course activities (behavioral participation), develops students’ positive emotions (emotional participation) in the course and activities, and gives students confidence to complete challenging tasks (cognitive participation) (Furrer and Skinner, 2003; Reeve, 2013; Ruzek et al., 2016; Vollet et al., 2017). Competency support from teachers is represented by academic support. When teachers concern about students’ studies, assigning learning activities that are appropriate to their abilities, provide them with instructional assistance and guidance and informational feedback in time, encourage them to do their utmost to succeed in their studies, students will perceive the competency support from teachers, this will give students confidence and enhance their engagement in learning. In conclusion, the hypothesis is proposed that basic psychological needs satisfaction plays a mediating role in the relationship between perceived teacher support and student engagement.

### 2.3. Learning drive as a mediator

What has always inspired students to study actively, and independently and achieve the corresponding goals is the core problem actively explored in pedagogy and educational psychology. The fundamental learning motivation should not be the coercion and suppression from the external environment, nor the external social normative motivation such as meeting the requirements of teachers, the expectations of elders and peer comparison, but the internal driving force of the pursuit of learning interest, self-improvement and self-development. Drive is the internal force that drives an organism to produce a certain behavior (Wang et al., 2017). The learning drive is an important personal resource that can facilitate student participation in their studies. During the learning process, students may be influenced by multiple motivational structures, such as an interest in a subject, whether the subject is interesting, the utility of the subject, the importance of the subject, and self-efficacy for the subject and long-term goals. This study considers that learning drive is the summary of the psychological factors related to learning, which includes both internal motivation, namely direct motivation, representing students “want to learn” and behavioral opportunity, namely self-efficacy, representing students “enable to learn”, task value, representing “worth learning”. Internal motivation, task value and self-efficacy are three indispensable dynamic factors in students’ learning process. So, this study defines learning drive as a multifaceted, positive motivative state including task value (personal interest), a high level of self-efficacy, and motivation to complete rich learning experience for different intrinsic reasons (Phan and Ngu, 2021). Task value is students’ evaluation of the interest, importance, or usefulness of a task. Self-efficacy is the expectation of success, meaning the individual’s prediction of her or his ability to perform specific tasks in the future. The learning intrinsic motivation is defined as the degree to which students participate in tasks for reasons of challenge, spontaneous interest and enjoyment, curiosity, and mastery. High level of task value perception and self-efficacy and internal motivation will promote students’ positive learning experience, and then promote students’ learning status and student engagement. Learning drive can make students actively identify with learning in ideology, ignite students’ desire to learn, make students have learning enthusiasm, initiative and persistence, and can make students consciously and continuously participate in the learning process. Learning drive is the internal motivation to maintain and promote students’ independent and conscious learning, and constantly improve their ability (Huo, 2020). It is the inner motivation for students to decide to invest to study and continue. The key to realize effective learning lies in making students have the internal driving force of learning and stimulate the lasting motivation for students to learn, which is exactly what the current higher vocational students lack. In view of this, the study introduces learning drive to investigate the relationship between perceived teacher support and student engagement, and assumes that learning drive plays a mediating role between perceived teachers’ support and student engagement.

### 2.4. Basic psychological needs satisfaction and learning drive as the chain mediators

When basic psychological needs are satisfied, internal motivation, social development, and happiness are promoted, and students will have higher academic engagement and achievement. Faye and Sharpe (2008) believed that academic environments which promote the satisfaction of three needs of autonomy, competence and relatedness can lead to self-determined behaviors (i.e., autonomous motivation). Niemiec and Ryan (2009) found that in classroom environments that support autonomy, competence and relatedness, students tend to be more internally motivated, more willing to engage in less interesting tasks, more autonomous in learning, and attach importance to learning activities. Therefore, learners can get higher quality of learning outcomes. Students with high self-efficacy are willing to make more efforts, often can continue to fulfill difficult tasks. Learning self-efficacy can affect student engagement among higher vocational college students (Yu, 2019). Task value positively affects students’ attitude towards learning, student engagement, and their academic achievement (Bandura, 1986). Through the correlation analysis, Gong et al. (2017) found that self-efficacy and task value were significantly and positively correlated with student engagement. The more students feel the importance, utility value and interest of learning tasks, the more inclined they are to experience the positive emotions in the learning process, and invest more enthusiasm and effort. Sansone and Harackiewicz (2000) believed that learning behaviors occur in a certain social environment. When students perceive the sense of autonomy, competence and relatedness from the environment, it will stimulate their internal motivation, and then encourage them to invest in learning tasks and achieve good academic performance. In conclusion, the hypothesis is proposed that the basic psychological needs satisfaction and the learning drive plays a chain intermediary role in the relationship between perceived teacher support and student engagement.

### 2.5. Optimistic attributional styles for explaining positive events as a moderator

Attribution is an individual’s perception of the causes of others or their behaviors or event results, it is the way that an individual explains causal relationships. Attribution style refers to the individual’s attribution cognitive style and cognitive tendency, which is a unique way of thinking that individuals habitually explain the reasons for different events in their learning and life (Abramson et al., 1978). In this study, from the perspective of positive psychology, the attribution style was divided into optimistic and pessimistic attribution style. Optimism attribution includes optimism after positive events and optimism after negative events. People with optimistic attributional styles (OAS) habitually treat good events as caused by internal (for them), stable, i. e., global (and global) factors rather than a specific part, and adverse events as external, unstable, and specific factors (Peterson et al., 1982). People with optimistic attribution style (optimistic attributional styles, OAS) habitually treat good events as caused by internal (to them), stable (i. e., permanent but not variable), and global (i. e., affecting various parts of life) factors, while adverse events are caused by external, unstable, and specific factors (Peterson et al., 1982).

Optimistic attribution style for explaining positive events (OAS_P) involves explaining good results (such as good performance in tests, competitions) is due to internal, global, and stable factors which is important for predicting academic and achievement outcomes. Optimistic assessment of positive events is able to provide positive emotions that help activate and sustain broadening and the construction processes in the learning environment. Moreover, OAS_P cognitively links past effort spending to future success to future success, and it can actively strengthen academic efforts.

Several studies have found that OAS_P are more predictive of academic performance than optimism following negative events (OAS_N) (Yates et al., 1995; Gordeeva et al., 2020). Houston (2016) even found that among school children, OAS_P predicted academic performance, while OAS_N did not. Therefore, this study focused on the optimistic attribution style for explaining positive events (OAS_P). Students with OAS_P are likely to make an internal, stable, global, and controllable attribution of past success (e. g., “I am competent and intelligent”). Optimistic thinking can enhance individuals’ positive expectations for future success, help people maintain motivation and experience positive emotions such as self-confidence and self-esteem. It can also stimulate perseverance after failure, make use of positive coping strategies to solve problems, thus improve learning motivation, and help to promote student engagement and improve academic achievement. According to attribution theory, students’ motivation is influenced by how students explain the success and failure of a field, some causal attribution likely to maintain or even increase motivation, while others reduce motivation (Graham, 2020). The researchers found that when the task was presented to the individual in the form of autonomous support, the individual senses that the behavior is highly autonomous, the internal motivation is activated, and the autonomous behavior occurs (Deci et al., 1981). However, there are significant individual differences in task learning effects, and perhaps this is related to individual attribution to learning effects (Ryan and Connell, 1989). In conclusion, this study puts forward the hypothesis that OAS_P plays a moderating role between basic psychological needs satisfaction and learning drive, and between learning drive and student engagement.

**Figure 1:**
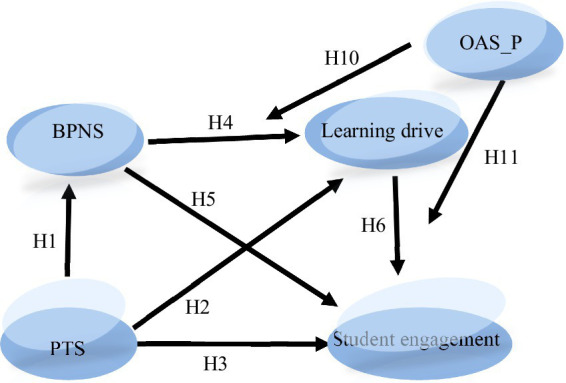
A moderated mediation model of perceived teacher support and student engagement among higher vocational students. PTS, Perceived teacher support; BPNS, basic psychological needs satisfaction.

Based on the above research results, this research puts forward the following research hypotheses, and constructs the concept model of Figure 1, so as to explore the influencing mechanism of higher vocational students’ perceived teacher support on student engagement. Research hypotheses are as follows:

*H1*: Perceived teacher support positively predicts basic psychological needs satisfaction.

*H2*: Perceived teacher support positively predicts learning drive.

*H3*: Perceived teacher support positively predicts student engagement.

*H4*: Basic psychological needs satisfaction positively predicts learning drive.

*H5*: Basic psychological needs satisfaction positively predicts student engagement.

*H6*: Learning drive positively predicts student engagement.

*H7* (*H1* → *H4*): Perceived teacher support positively predicts learning drive through basic psychological needs satisfaction.

*H8* (*H2* → *H6*): Perceived teacher support positively predicts student engagement through learning drive.

*H9* (*H1* → *H4* → *H6*): Perceived teacher support positively predicts student engagement through basic psychological needs satisfaction and learning drive.

*H10*: Optimistic attribution style moderates the relationship of basic psychological needs satisfaction and learning drive.

*H11*: Optimistic attribution style moderates the relationship of learning drive and student engagement.

The conceptual model of this study is shown in Figure 1. The figure shows the relationship between the latent variables in the model, but does not show the relationship between the measured variables and the latent variables (namely the measured model).

## 3. Methods

### 3.1. Participants

This study adopted a convenient sampling method. A total of 1,136 students participated in this study, they came from 6 vocational and technical colleges in four Chinese provinces, including Zhejiang province, Tianjin, Gansu province and Shanxi province. The questionnaire survey was conducted using a combination of online and offline methods. Part of the questionnaire was distributed online on Wechat or QQ, and part of the paper version of the questionnaire was distributed offline. Since through the online questionnaire, we can see the answer time of the participants, if the answer time is too short, we have reason to believe that the participant did not read the items carefully and we can doubt the accuracy of his (her) answers. If all the answers to all items are same, we can believe that the participant did not treat the items seriously and his (her) responses were not credible. So, when eliminating the invalid questionnaires, we removed not only the questionnaires with missing values and regular answers, but also the questionnaires with too short answer time and all the same answers. Eventually,1,031 questionnaires were deemed valid with a response rate of 90.75%. Of the total participants, 279 were freshmen (27.1%), 354 are sophomores (34.3%) and 398 are juniors (38.6%). In terms of their gender, 626 are males (60.7%) and 405 are females (39.3%).

### 3.2. Measures

Based on the qualitative interview and the mature scale at home and abroad, a questionnaire of student engagement among higher vocational students was produced. The questionnaire includes four scales: the students’ basic demographic information and perceived teacher support, basic psychological needs satisfaction, learning drive and student engagement. Confidence validity of the scale was tested using SPSS 26.0 and Amos 24.0 software. Hypothesis test was accomplished by Amos 24.0 software.

#### 3.2.1. Perceived teacher support scale

Perceived teacher support was measured by the revised teacher support scale designed by Chi (Chinese version) (2017). Based on the Self-Determination Theory, Chi divided teacher support into three dimensions: autonomy support, competence support and emotion support. The scale has three subscales, and it comprised a total of 13 items. Among them, autonomy support has 5 items, emotion support has 4 items and competency support has 4 items. In this study, each item is rated by a 7-point Likert-type (1 as totally inconsistent, 7 as fully consistent). A higher score represents a higher level of perceived teacher support. The Cronbach’s α of this scale is 0.879.

#### 3.2.2. Basic psychological needs satisfaction scale

The basic psychological needs was assessed by the Basic Needs Satisfaction in General Scale (BNSG-S) (Johnston and Finney, 2010), including autonomy (7 items), competence (6 items) and relatedness (8 items) three sub-scales, a total of 21 items. In this study, higher vocational students were asked to point out how true they thought each statement was about their lives, scoring each item using a 7-point Likert-type (1 = Not at all true, 7 = extremely true to me). A higher average score indicates a higher basic psychological needs satisfaction. The Cronbach’s α of this scale is 0.909.

#### 3.2.3. Learning drive scale

In this study, the learning drive scale includes learning intrinsic motivation, task value, and self-efficacy three sub-dimensions. The two sub-dimensions of learning intrinsic motivation and self-efficacy were referred to the MSLQ (Motivated Strategies for Learning Questionnaire) developed by Pintrich et al. (1991), which is a valid and reliable questionnaire (Paulsen and Feldman, 2007). The students rated each item with 7-point Likert-type from “1” (completely inconsistent) to “7” (completely consistent). In order to adapt to the general situation, the corresponding items were modified. For example, the item “in this course, I prefer to learn the challenging contents because I can learn something new” was changed to “I prefer to learn the challenging contents because I can learn something new.” The task value sub-dimension was designed according to the researches of task value sub-dimensions in MSLQ scale (Hulleman et al., 2008, 2010; Johnson and Sinatra, 2013). The task value sub-dimension in MSLQ scale is used for the students’ cognition and evaluation of the interest, importance, and usefulness of a certain course task. Hulleman et al. (2008, 2010) had considered the two dimensions of intrinsic value and utility value. Johnson and Sinatra (2013) focused on the two dimensions of utility value and achievement value, both were measured by 6 items, it enriched the MSLQ scale. In this study, there are 5 items of intrinsic value sub-dimension and 5 items of utility value sub-dimension, and 3 items of achievement value sub-dimension. Students rated each item on a 7-point Likert-type (1 = completely inconsistent, 7 = completely consistent). The Cronbach’s α of this scale is 0.757.

#### 3.2.4. Student engagement scale

The student engagement scale was designed according to the studies of Skinner et al. (2008) and Lam et al. (2014). The scale has three subscales: behavioral engagement (with 6 items), emotional engagement (with 5 items), and cognitive engagement (with 7 items). The measurement was performed using a 7-point Likert-type. The Cronbach’s α of this scale is 0.881.

#### 3.2.5. OAS_P scale

The OAS_P scale was designed on the basis of the academic achievement sub-scale of Multidimensional-Multiattributional Causality Scale (MMCS), which was compiled by Lefcourt et al. (1979). The academic achievement sub-scale contains four dimensions of competence, effort, background (context) and luck. We referred to the items about success experience in competence and effort dimensions. Because OAS_P tends to interpret good results (e. g., good performance in tests, competitions) as persistent factors such as stability, global, and internal factors, like competence and efforts. This scale consists of seven items using a 7-point Likert-type. The Cronbach’s α of this scale is 0.904.

## 4. Results

### 4.1. Common method bias test

In this study, the Harman single factor test was used to test for common method bias. All items from the five sub-scales of perceived teacher support scale, basic psychological needs satisfaction scale, learning drive scale, student engagement scale and OAS_P scale were subjected to an exploratory factor analysis in SPSS 26.0, choosing non-rotated principal component analysis. The explained variance percentage of the first common factor is generally required as less than 30% (strict), less than 40% (ideal), and less than 50% (acceptable) (Podsakoff et al., 2003). The results show that there are five factors with eigenvalue greater than 1 extracted, and the cumulative explanatory variance was 69.942%, among which the explanatory variance of the first factor was 33.974%, which was less than the 40% critical standard. Therefore, there was no significant a common method bias in the sample data of this study.

### 4.2. Descriptive statistics and correlation analysis of each variable

In order to check the possible correlations among the variables, the correlation analysis was conducted on the factors of perceived teacher support, basic psychological needs satisfaction, learning drive, student engagement and OAS_P, and the results are shown in Table 1. Pearson correlation coefficient, mean and standard deviations are illustrated in Table 1. The mean of variables ranged from 3.76 (OAS_P) to 5.12 (perceived teacher support). The Pearson correlation coefficient was statistically significant, ranging from 0.279 to 0.461, with a moderate size. The correlation matrix of each latent variable shows the correlation between the factors: (1) Perceived teacher support was significantly associated with basic psychological needs satisfaction (*r* = 0.377, *p* < 0.01), perceived teacher support was significantly associated with learning drive (*r* = 0.461, *p* < 0.01), perceived teacher support was significantly associated with student engagement (*r* = 0.377, *p* < 0.01), and perceived teacher support was significantly associated with OAS_P (*r* = 0.279, *p* < 0.01), (2) The basic psychological needs satisfaction showed a significant positive correlation with learning drive (*r* = 0.388, *p* < 0.01), the basic psychological needs satisfaction showed a significant positive correlation with student engagement (*r* = 0.216, *p* < 0.01), and the basic psychological needs satisfaction showed a significant positive correlation with OAS_P (*r* = 0.318, *p* < 0.01), (3) There was a significant positive correlation between learning drive and student engagement (*r* = 0.320, *p* < 0.01). There was a significant positive correlation between learning drive and OAS_P (*r* = 0.332, *p* < 0.01), and (4) There was a significant positive correlation between student engagement and OAS_P(*r* = 0.385, *p* < 0.01).

**Table 1:** Correlation, mean (*M*), and standard deviation (SD) of each latent variable.

	1	2	3	4	5
1 perceived teacher support	1				
2 basic psychological needs satisfaction	0.377^**^	1			
3 learning drive	0.461^**^	0.388^**^	1		
4 student engagement	0.377^**^	0.216^**^	0.320^**^	1	
5 OAS_P style	0.279^**^	0.318^**^	0.332^**^	0.385^**^	1
*M*	5.121	5.294	4.727	4.311	3.759
SD	0.839	1.042	0.948	1.112	1.421

** indicates significant correlation at 0.01 (double tail).

### 4.3. The effect of perceived teacher support on student engagement

#### 4.3.1. Model-fit degree test

**Figure 2:**
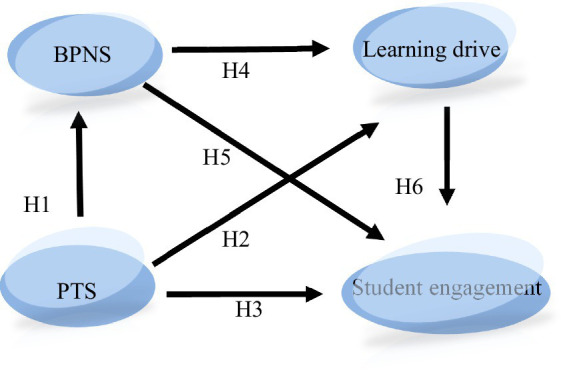
A mediation model of perceived teacher support and student engagement among higher vocational students. PTS, Perceived teacher support; BPNS, Basic psychological needs satisfaction.

On the basis of the correlation analysis of the potential variables, this study adopts the structural equation model in Figure 1 to investigate the mediating effects of the basic psychological needs satisfaction and the learning drive to student engagement, and chain mediating effect of the basic psychological needs satisfaction and the learning drive to student engagement. The model is shown in Figure 2.

In this study, the maximum likelihood estimation method and AMOS 24.0 software was used to test the Figure 1 model, and the fitting indexes between the data and the model are *χ*^2^ = 135.315, df = 48, *χ*^2^/df = 2.819 < 3, SRMR = 0.0365 < 0.08, RMSEA = 0.031 < 0.08, CFI = 0.975 > 0.90, NFI = 0.912 > 0.90. It shows that the model has a very good fitting degree. So the overall fit of the model is good.

#### 4.3.2. Direct effect test

**Figure 3:**
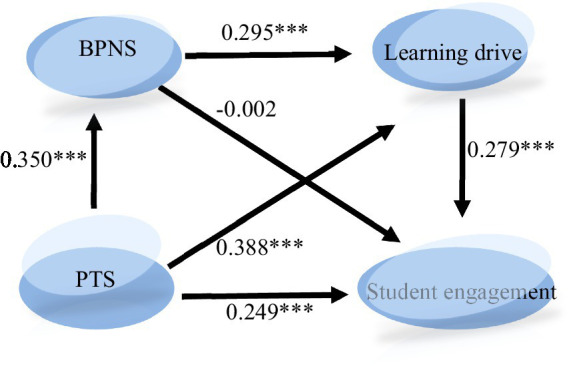
Model path diagram and standardized estimates. PTS, Perceived teacher support; BPNS, Basic psychological needs satisfaction.

According to the results shown in Figure 3 and Table 2, the standardized path coefficients of the hypothesis H1, H2, H3, H4, and H6 are, respectively, 0.350, 0.388, 0.249, 0.295, and 0.279, and the *p*-values are less than 0.05, so H1, H2, H3, H4, and H6 are supported. While the standardized path coefficients of hypothesis H5 is −0.002, *p* = 0.974 > 0.05, so the hypothesis H5 is non-supported.

**Table 2:** The test result of path relationship.

Hypothesis path	Unstd	S.E.	Value of *Z*	Std.	Value of *p*	Supported?
H1 perceived teacher support → basic psychological needs satisfaction	0.430	0.057	7.544	0.350	***	Yes
H2 perceived teacher support → Learning drive	0.311	0.047	6.617	0.388	***	Yes
H4 basic psychological needs satisfaction → Learning drive	0.192	0.036	5.333	0.295	***	Yes
H3 perceived teacher support → student engagement	0.271	0.062	4.371	0.249	***	Yes
H5 basic psychological needs satisfaction → student engagement	−0.001	0.046	−0.022	−0.002	0.974	No
H6 learning drive → student engagement	0.379	0.095	3.989	0.279	***	Yes

#### 4.3.3. Mediating effects test

In AMOS 24.0 software, the maximum likelihood estimation method was chosen, and Bootstrapping by repeated sampling 5,000 times is used. The significance test of the mediating effect of perceived teacher support and learning input was tested under the setting condition of 95% confidence interval. The results of the mediating effect test are shown in Table 3. Whether the effect was significant is judged by the 95% confidence interval. If the 95% confidence interval includes 0, the effect is non-significant. Otherwise the effect is significant. Therefore, the path perceived teacher support → basic psychological needs satisfaction → student engagement is not significant, hypothesis H7 is not supported, the path perceived teacher support → learning drive → student engagement and the path perceived teacher support → basic psychological needs satisfaction → learning drive → student engagement are significant, hypothesis H8 and H9 is supported.

**Table 3:** The test results of the mediating effect.

Path relationship	Point estimate	Product of coefficient		Bootstrapping 5,000 times 95% CI			
		SE	Value of *Z*	Bias-corrected		Percentile	
				Lower limit	Upper limit	Lower limit	Upper limit
*Mediating effect test*							
IE1	−0.001	0.023	−0.043	−0.045	0.047	−0.044	0.049
IE2	0.118	0.045	2.622	0.048	0.223	0.049	0.224
IE3	0.031	0.013	2.385	0.013	0.066	0.012	0.062
*Mediating effect comparison*							
C1	−0.118	0.052	−2.269	−0.236	−0.030	−0.236	−0.030
C2	−0.032	0.030	−1.067	−0.105	0.019	−0.097	0.025
C3	0.086	0.042	2.048	0.026	0.190	0.025	0.188
*Mediating effect ratio*							
P1	−0.004	0.183	−0.022	−0.423	0.287	−0.399	0.295
P2	0.793	0.138	5.746	0.515	1.040	0.525	1.055
P3	0.211	0.097	2.175	0.096	0.501	0.085	0.447

IE1 stands for the path perceived teacher support → basic psychological needs satisfaction → student engagement, IE1 = H1*H5, IE2 stands for the path perceived teacher support → learning drive → student engagement, IE2 = H2*H6, IE3 stands for the path perceived teacher support → basic psychological needs satisfaction → learning drive → student engagement, IE3 = H1*H4*H6. TIE represents the total indirect effect, TIE = IE1 + IE2 + IE3. C1, C2, and C3 indicate the comparison between mediating effects, respectively C1 = IE1−IE2, C2 = IE1−IE3, C3 = IE2−IE3. P1, P2, and P3 indicate the mediation effect ratio, respectively, P1 = IE1/TIE, P2 = IE2/TIE, and P3 = IE3/TIE.

#### 4.3.4. Moderation effect test

In this study, the analysis of the moderation effect model used the PROCESS 3.4 plugin in SPSS 26.0. Referring to the Bootstrap method proposed by Hayes, the model 58 was chosen, and repeated sampling 5,000 times is used, with 95% confidence interval. As can be seen from Table 4, OAS_P style has a significant moderation effect between the basic psychological needs and learning drive (the first half of the mediation effect), and between learning drive and student engagement (the second half of the mediation effect).

**Table 4:** The test results of the moderation effect.

dependent variable	interactive items	*R*^2^-change	Value of *p*	LLCI	ULCI	Supported?
Learning drive	Basic psychological needs satisfaction*OAS_P	0.016	0.002	−0.117	−0.028	Yes
Student engagement	Learning drive*OAS_P	0.017	0.001	0.040	0.162	Yes

**Figure 4:**
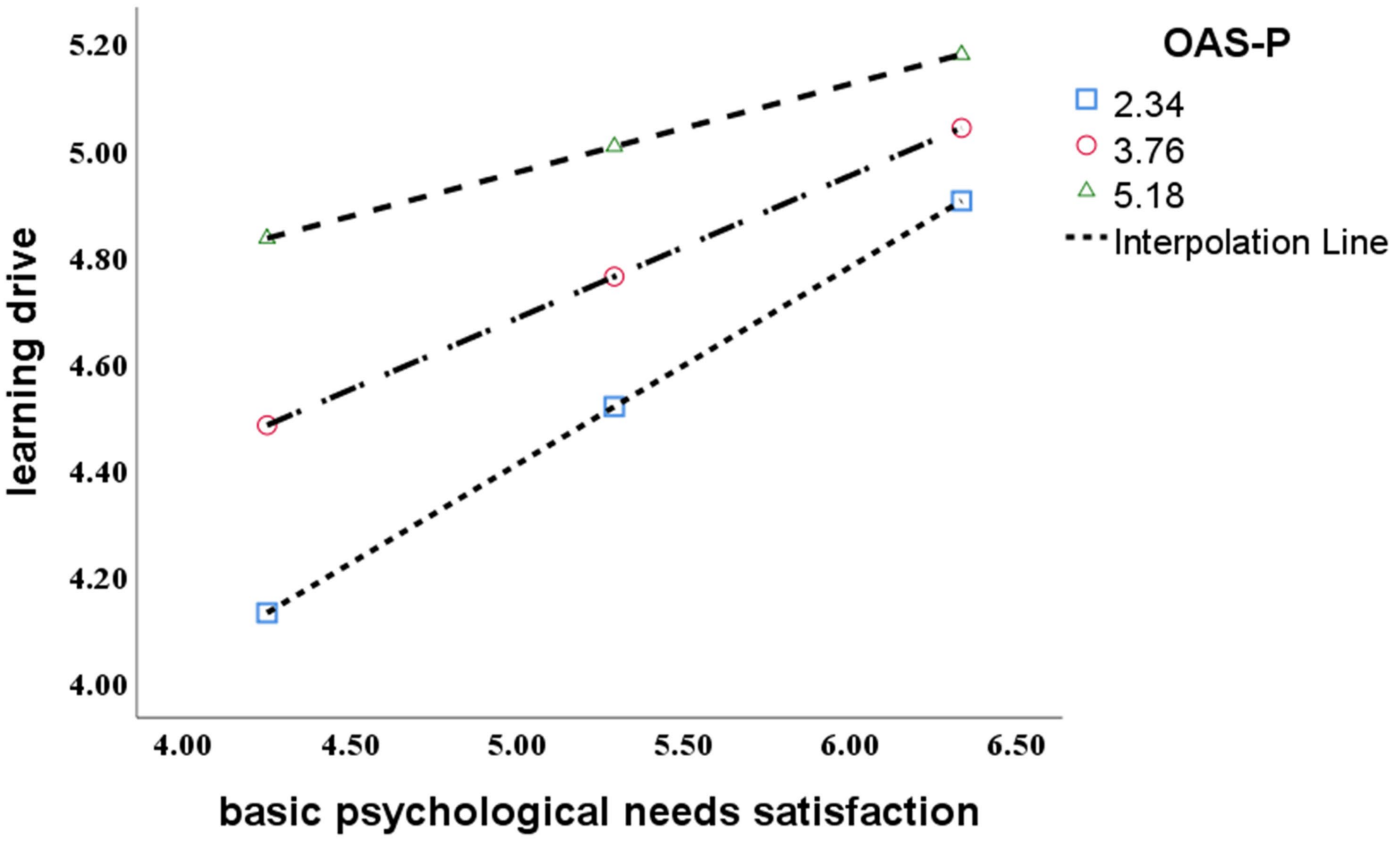
The moderation effect of OAS_P on the basic psychological needs satisfaction and student engagement.

**Figure 5:**
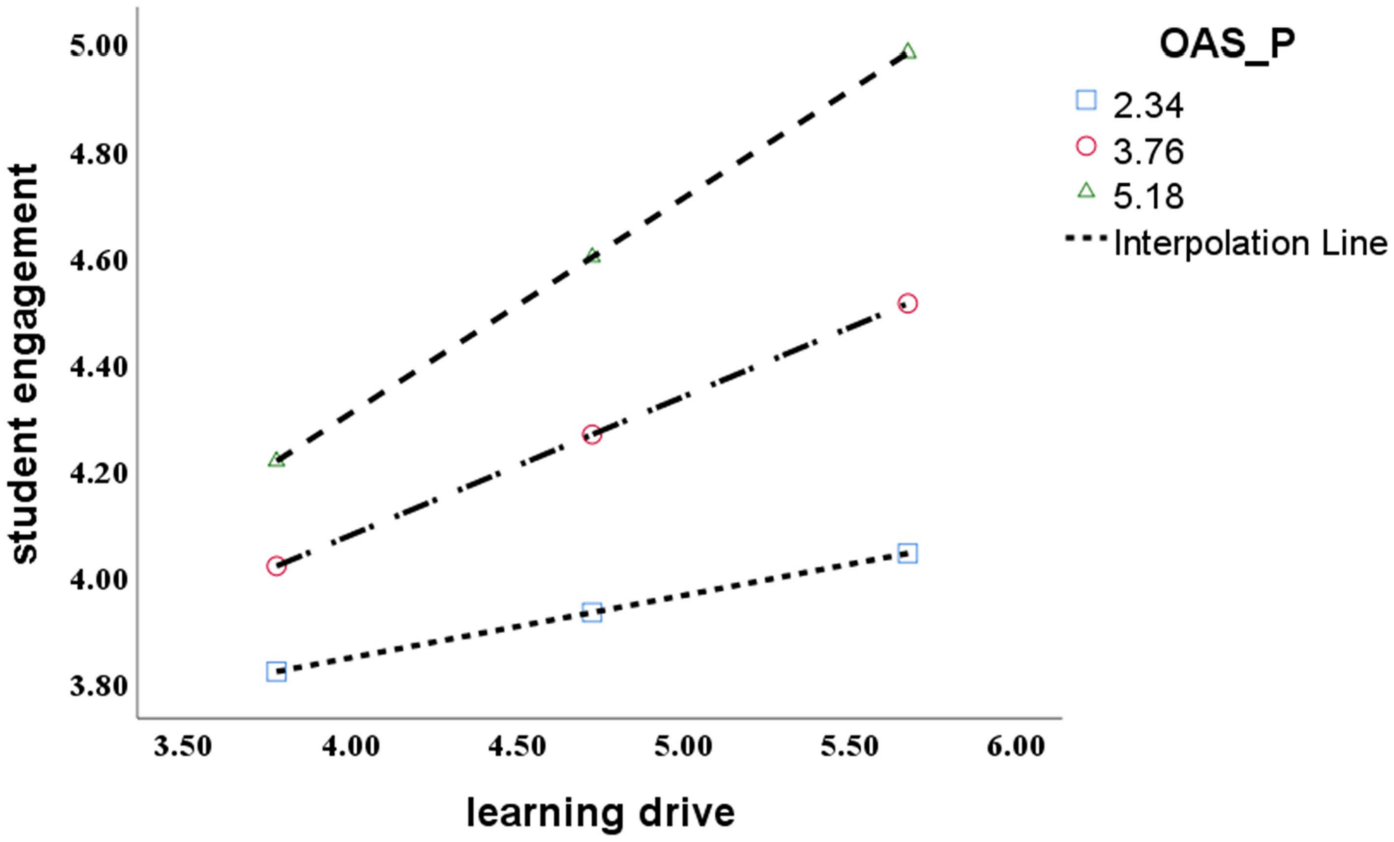
The moderation effect of OAS_P style on learning drive and student engagement.

In order to further analyze the moderation effect of OAS_P, OAS_P were grouped by a mean of ±1 standard deviation, representing the high, medium and low interference variables. Above one standard deviation is the higher OAS_P style group and below one standard deviation is the lower OAS_P style group. The regression analysis of the basic psychological needs satisfaction and learning drive was conducted, the fit regression line was obtained under different levels of OAS_P style, forming a simple slope effect map, as shown in Figure 4. The results show that for the higher vocational students with high and lower OAS_P style, the basic psychological needs satisfaction had a significant impact on the learning drive, but for the lower vocational students with low OAS_P style group, the basic psychological needs satisfaction had a greater impact to promote the learning drive, and hypothesis H10 is verified. The regression analysis of learning drive and student engagement was conducted, the fitted regression line was obtained under different levels of OAS_P style, forming a simple slope effect map, as shown in Figure 5. The results show that for the higher vocational students with high and low OAS_P style, learning drive had a significant impact on student engagement, but for the higher vocational students with low OAS_P style group, learning drive had a greater impact to promote student engagement, and hypothesis H10 is verified.

## 5. Conclusion

Based on some classical theories, such as constructivism learning theory, Bronfenbrenner’s ecosystem theory and self-determination theory, we take the relationship between environment-individual-student engagement as the main line, higher vocational students as the research object, build a structural equation model, conduct a questionnaire survey to explore the relationship between perceived teacher support, basic psychological needs satisfaction, learning drive and student engagement. Meanwhile, the mediating role of basic psychological needs satisfaction and learning drive, and moderation effect of OAS_P style are studied. The findings showed that (1) Perceived teacher support has significant positive effects on basic psychological needs satisfaction, learning drive and student engagement; Basic psychological needs satisfaction has a significant positive impact on learning drive; learning drive has a significant positive impact on student engagement; However, the impact of basic psychological needs satisfaction on student engagement is not significant. This is not consistent with the views in the aforementioned literature. This study believes that only the individual learning activities can be stimulated and maintained to promote individual student engagement when the basic psychological needs satisfaction is converted into learning needs, namely, learning drive. (2) Learning drive partially mediates the relationship between perceived teacher support and student engagement. Basic psychological needs satisfaction and learning drive have a multiple chain mediating effect between perceived teacher support and student engagement. (3) OAS_P style significantly moderates the relationship between basic psychological needs satisfaction and learning drive, learning drive and student engagement.

## 6. Limitations

There are some limitations in this study. First, This study used a cross-sectional study design, with the data collected only found to be cross-sectional, which can only reveal correlations between factors, and the obtained results can not be used to infer causal relationships between factors. In subsequent studies, a longitudinal design can be used in longitudinal studies to reveal whether these factors have an interactive nature and a causal relationship between these factors. Second, all the variables in the present study were measured in a self-reported manner which is vulnerable to subjective factors and social expectation effects. In the follow-up research, the data can be comprehensively collected in relatively objective ways such as observation and teacher report.

## 7. Suggestions

Lin et al. (2012) believes that higher vocational students have lower self-efficacy, lower interest in learning, and unclear learning goals, and some of them can not be correctly attributed to them. In the teaching process, teachers should pay attention to their learning psychology, provide them with various support and encouragement and beneficial guidance, stimulate their learning drive, help them form a positive and optimistic attribution style, and make them actively participate in learning and school life.

### 7.1. Provide students with timely encouragement and various beneficial guidance

Supportive environment is beneficial to promote the basic psychological needs satisfaction. In addition to mastering certain theoretical knowledge, higher vocational students need to master more practical skills, so they need more training. During the training, there is more interaction between students and teachers. Teachers have more influence on students’ learning. Teachers’ encouragement and corresponding guidance is very important. When students encounter difficulties, timely encouragement, suggestions and corresponding guidance can reduce students’ anxiety, avoid negative emotions, such as loose confidence, can make students more enthusiasm and more responsible for their learning.

### 7.2. Stimulate students’ learning drive

Xu et al. (2019) found that 90.22% of the surveyed higher vocational college students believed that “Reasons to study hard” is to find a good job after graduation; 85.67% thought that “What causes you to be interested in learning” is to learn what is useful to them, and 68.9% thought it was the interest of learning content. So the interesting and value of the learning tasks are very important for students. In the teaching practice, teachers can make good use of ingenious instructional design, vivid explanation like metaphors, video demonstration, virtual simulation and so on to make the learning content interest. Teachers can use something similar to “Can this learning activity help you develop an important skill?,” “Is this learning task important?,” “Is this task useful for your future career pursuit? “, explain why learning activities are useful and important for students’ future careers with convincing and meaningful reasons, to induce students to recognize the usefulness, utility and importance of the learning task, improving students’ value cognition, curiosity and interest of learning tasks, especially the uninteresting tasks. Self-Efficacy is an essential motive to learn. However, Ma (2021) found that the self-efficacy level of higher vocational students is just medium, not reaching a good level. In order to promote students’ sense of self-efficacy, teachers can create more opportunities for students to succeed, let students make full use of good grades and their own small success; Set up learning models or examples for higher vocational students to follow, which can mobilize students’ emotional state and drive students to learn actively.

### 7.3. Conduct attribution training for students to form a positive and optimistic attribution style

Ding and Wang (2015) believe that most of the higher vocational students who choose to study in higher vocational colleges due to the failure in the college entrance examination, they often attribute their unsatisfactory academic performance to the stable internal and external factors such as their weak learning ability and the difficulty of the examination, and did not establish the correct attribution. This bad tendency is easy to lead to students to self-abandonment in learning, lack of behavioral persistence, and form a vicious circle.

Through attribution training, expose students’ attribution style, guide students to analyze their unreasonable attribution, help them clarify the unreasonable attribution, strengthen the self-awareness training of appreciation and pleasant to accept self, establish a correct view of attribution, form a positive and optimistic attribution style, and it can effectively stimulate the learning drive and student engagement among higher vocational students. A lamed traveler should get out betimes. Practice makes perfect. Encourage students to think optimistically, believe in the significance of hard work, and believe that hard work can influence future events, so that they will be motivated to learn and actively participate into the learning process.

## Declarations

In this study, all participants were informed that participation was voluntary.

## Data availability statement

The original contributions presented in the study are included in the article/supplementary material, further inquiries can be directed to the corresponding author.

## Author contributions

XX and ZW: conceptualization. ZW: methodology, validation, supervision, project administration, and funding acquisition. XX: software, data curation, and writing-original draft preparation. XX and DW: investigation. DW: writing-review and editing. All authors contributed to the article and approved the submitted version.

## Funding

This research was funded by the Major Social Science Project of Tianjin Municipal Education Commission, China (grant number 2022JWZD37).

## Conflict of interest

The authors declare that the research was conducted in the absence of any commercial or financial relationships that could be construed as a potential conflict of interest.

## Publisher’s note

All claims expressed in this article are solely those of the authors and do not necessarily represent those of their affiliated organizations, or those of the publisher, the editors and the reviewers. Any product that may be evaluated in this article, or claim that may be made by its manufacturer, is not guaranteed or endorsed by the publisher.
